# Development of a Colloidal Gold Immunochromatographic Strip for the One-Step Evaluation of the Total Content of Rhein and Aloe-Emodin in Rhubarb

**DOI:** 10.1155/2022/7067245

**Published:** 2022-04-26

**Authors:** Ping Sun, Xin-Peng Li, Jie Xin, Tao Xue, Bo Zhang, Yan-Juan Liu

**Affiliations:** School of Pharmacy, Linyi University, Linyi 276000, China

## Abstract

In this study, a colloidal gold immunochromatographic strip was developed for simultaneous detection of rhein and aloe-emodin in rhubarb. The cutoff value, defined as the lowest concentration for which the test line was invisible on the strip, was 50 ng mL^−1^ for both rhein and aloe-emodin. By contrast, the cutoff value for emodin was 2,000 ng mL^−1^. No competitive inhibition was observed up to 5,000 ng mL^−1^ of physcion, chrysophanol, sennoside A, sennoside B, or rhaponticin. Semiquantitative analyses of the total contents of rhein and aloe-emodin in raw drug materials via our colloidal gold immunochromatographic strips produced results agreeable with those determined by HPLC. Taken together, our findings suggest that the implementation of our colloidal gold immunochromatographic strips provides a rapid one-step method for estimating the total contents of rhein and aloe-emodin, which may represent a powerful tool for quality control of rhubarb.

## 1. Introduction

Rhubarb, derived from the roots and rhizomes of three species of plants—including *Rheum palmatum* L., *Rheum tanguticum* Maxim. ex Balf., and *Rheum officinale* Baill—has been used as a traditional Chinese medicine for thousands of years in China and is now used worldwide [1]. Although rhubarb is best known for its purgative activity, it has also been confirmed to possess antibacterial [2], anti-inflammatory [3–5], antidiabetic [6, 7], renal-protective [8], and photoprotective [9] properties. However, the quality of rhubarb is greatly affected by genetic and/or environmental factors. Therefore, it is necessary to establish a simple and rapid quality control method for rhubarb.

**Figure 1:**
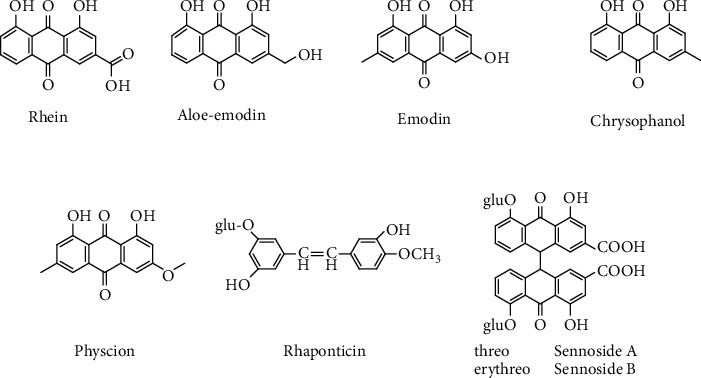
Chemical structures of anthraquinones and its analogues in rhubarb.

Anthraquinones (Figure 1)—including rhein, aloe-emodin, emodin, physcion, and chrysophanol—are the main active substances in rhubarb. Additionally, anthraquinones have been used as quality control markers of rhubarb, as documented in the *Chinese Pharmacopoeia* (2020 edition) [1]. Furthermore, a previous study has shown that rhein and aloe-emodin represent the two key species-specific markers of rhubarb [10]. Thus, in our study, we aimed to develop a rapid analysis method for the determination of rhein and aloe-emodin in raw drug materials.

To date, a variety of analytical methods—including high-performance liquid chromatography (HPLC) [11], ultra-performance liquid chromatography (UPLC) [12], capillary electrophoresis (CE) [13], ^1^H nuclear magnetic resonance (^1^H NMR) [14], and near-infrared spectroscopy (NIS) [15]—have been used for the quantitative detection of anthraquinones in rhubarb. However, these methods have shortcomings such as being tedious, time-consuming, costly, and requiring sophisticated techniques. Compared with the above-mentioned methods, immunoassays, especially enzyme-linked immunosorbent assays (ELISAs) and immunochromatography, are simple, rapid, sensitive, high-throughput, and ecofriendly. Similarly, colloidal gold immunochromatographic (CGIC) strips are powerful tools that enable rapid and low-cost detection of specific contents and have been used as home-pregnancy test strips since they do not require any special training or instruments [16]. In our previous work [17], we developed an indirect competitive ELISA for rhein detection based on the monoclonal antibody, 1F8 (mAb1F8), against rhein. Additionally, Zhang et al. [18] developed a quantum-dot-based lateral-flow immunochromatographic strip (QD-LCS) for rapid detection of rhein. However, to the best of our knowledge, no form of immunochromatography has yet been established for simultaneous detection of rhein and aloe-emodin.

In the present work, a CGIC strip was developed for simultaneous detection of rhein and aloe-emodin. Specifically, we used our CGIC strips to analyze the total contents of rhein and aloe-emodin in different rhubarb samples and then verified these results via HPLC.

## 2. Materials and Methods

### 2.1. Materials

Rhein-bovine serum albumin (BSA) conjugate and mAb1F8, which recognizes both rhein and aloe-emodin, were produced in our laboratory according to our previous report [17]. The mAb1F8 was dialyzed against deionized water and lyophilized before using. Rhein and aloe-emodin were purchased from Push Bio-technology Co., Ltd. (Chengdu, China). Goat anti-mouse IgG and BSA were purchased from Sigma-Aldrich Chemical Co. (St. Louis, MO, USA). Colloidal gold (30 nm) and other raw materials required for our CGIC strips were provided by Shanghai Jieyi Biotechnology Co., Ltd. (Shanghai, China).

Three official rhubarb samples, namely, *Rheum palmatum* L., *Rheum tanguticum* Maxim. ex Balf., and *Rheum officinale* Baill., and two counterfeit species, *Rumex crispus* Linn. and *Rheum franzenbachii* Munt., were obtained from the National Institutes for Food and Drug Control (NIFDC; Beijing, China). Additionally, another five rhubarb samples were collected from different pharmacies in China.

### 2.2. Preparation of Colloidal Gold-mAb1F8 Conjugates

First, mAb1F8 powder was dissolved in double-distilled water and was then centrifuged (8,000 g) at 4°C for 10 min. The supernatant was collected, and the final concentration of mAb1F8, which determined by a nanodrop spectrophotometer (Tucson visual technology, Shanghai, China), was adjusted to 1 mg mL^−1^.

Before the colloidal gold was conjugated with mAb1F8, the optimal pH value of the colloidal gold reagent and the working concentration of mAb1F8 were verified by checkerboard titration.

The conjugation experiment was performed via the following steps. First, the colloidal gold reagent was adjusted to a pH of 8.0 with 0.2 M of K_2_CO_3_ solution. Second, 30 *μ*L of mAb1F8 solution (1 mg mL^−1^) was added to 10 mL of colloidal gold solution while stirring; the reaction was then maintained for 10 min. Third, 200 *μ*L of 10% (w/v) BSA (containing 0.1% NaN_3_) was added to the suspension. After stirred for 10 min, the mixture was centrifuged (8,000 g × 20 min) at 4°C. Fourth, the gold-mAb1F8 conjugate was collected from the bottom of the tube, redissolved with 1 mL of 0.01 M of phosphate buffer (PB, pH = 8.0), and finally incubated on a conjugate pad overnight at room temperature.

### 2.3. Preparation of Capture Reagents

Goat anti-mouse IgG (1 mg mL^−1^) and rhein-BSA (1 mg mL^−1^) were used as a control capture reagent and test capture reagent, respectively. Each of the two reagents was dispensed separately as lines on nitrocellulose-membrane strips (300 × 25 mm) via a dispenser (Jieyi Biotechnology, Shanghai, China). The control line was dispensed on the top side of the membrane, while the test line was dispensed on the bottom side. The two lines were spaced 0.5 cm apart from one another. The sprayed volumes for the lines were 1 *μ*L cm^−1^. After dispensing, the membrane was dried in an oven at 37°C for 30 min to immobilize the reagents.

### 2.4. Preparation of CGIC Strips

**Figure 2:**
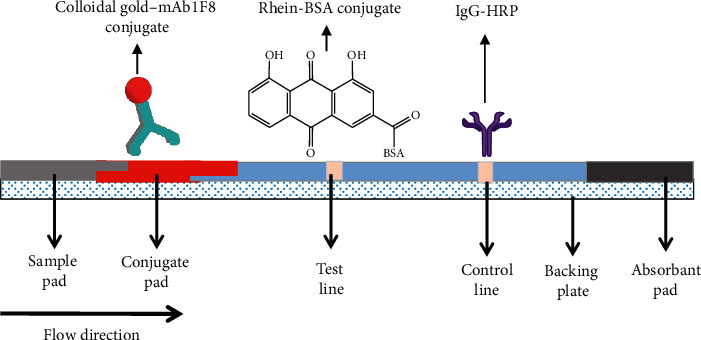
Assembly of CGIC strips.

A polyvinyl chloride (PVC)-backing plate was used for supporting each CGIC strip. A nitrocellulose membrane with capture reagents, colloidal gold-mAb1F8 conjugate pad, sample pad, and an absorbent pad was pasted onto the PVC plate. Their order, from top to bottom, was as follows: absorbent pad, nitrocellulose membrane, colloidal gold-mAb1F8 conjugate pad, and sample pad. These components overlapped each other by 1–2 mm each (Figure 2). After complete assembly, the plate was cut into strips (60 × 4 mm).

### 2.5. Evaluation of CGIC Strips: Cutoff Values and Specificities

Stock solutions of rhein and aloe-emodin were prepared in methanol (80 *μ*g mL^−1^) in gradient concentrations of rhein (200, 100, 50, 25, 12.5, 6.25, and 3.13 ng mL^−1^) and aloe-emodin (100, 50, 25, 12.5, 6.25, 3.13, and 1.56 ng mL^−1^) that were diluted in distilled water and used to confirm cutoff values of the CGIC strips. The cutoff value was defined as the lowest concentration for which the test line was invisible [19]. Specifically, 50 *μ*L of standard solutions was used to estimate the cutoff values of the CGIC strips. The results were then evaluated by the naked eye within 6 min at room temperature.

Furthermore, emodin, physcion, chrysophanol, sennoside A, sennoside B, and rhaponticin were tested for their specificities. Gradient concentrations (5,000, 2,500, 1,250, 625, 313, 156, and 78 ng mL^−1^, respectively) of physcion, chrysophanol, sennoside A, sennoside B, and rhaponticin were used for testing. However, the gradient concentrations of emodin were 4,000, 2,000, 1,000, 500, 250, 125, and 62.5 ng mL^−1^, respectively. All the compounds were dissolved in methanol and diluted with distilled water. Each sample was analyzed in triplicate.

### 2.6. Sample Extraction and Evaluation

Ten crude drug samples were powdered and dried in an oven at 60°C for 5 h. Next, 0.5 g of each of the powdered sample was extracted with 25 mL of methanol for 30 min in an ultrasonic bath [17, 20]. After centrifugation (8,000 g) for 20 min, the supernatant was collected for subsequent testing. Part of the supernatant was analyzed by CGIC strips after being diluted 50–3200 times with distilled water. The other part of the supernatant was analyzed directly by HPLC after being filtered through a 0.22-*μ*m membrane filter. Each sample was analyzed in triplicate.

### 2.7. HPLC

An ACCHROM S6000 system (Acchrom Tech, Beijing, China) was used for rhein and aloe-emodin analysis in crude drug samples. An Inertsil-ODS-3-column (250 × 4.6 mm, 5 *μ*m; GL Sciences, Japan) was used as the stationary phase. The mobile phase was composed of methanol and 0.1% phosphoric acid at a ratio of 80 : 20 (*v/v*). The flow rate was 1.0 mL min^−1^, the detection wavelength was 254 nm, and the injection volume was 10 *μ*L.

### 2.8. Storage of CGIC Strips

To determine the stability of CGIC strips, the strips were stored 2 weeks at 37°C and for 3 months at 4°C. After storage, their cutoff values for rhein and aloe-emodin were evaluated.

## 3. Results and Discussion

### 3.1. Development of Strip

As we have previously reported [17], the cross-reactivity of mAb1F8 with aloe-emodin is approximately 27%. This high cross-reactivity makes it possible to develop a CGIC strip for simultaneous determination of rhein and aloe-emodin contents.

Our CGIC strip assay that we developed in the present study was based on a competitive principle using mAb1F8 as a detector. After being added to the sample pad, standard solutions and test samples migrated upwards via capillary action to the colloidal gold-mAb1F8 conjugate pad. Furthermore, rhein and/or aloe-emodin were recognized by the colloidal gold-mAb1F8 conjugate pad and combined with it. When sufficient concentrations of rhein and/or aloe-emodin existed in a test solution, the test lines were invisible. On the contrary, the test lines were visible if there were not sufficient concentrations of rhein and/or aloe-emodin present in a test solution. The color intensity of the test lines was attenuated as a function of the concentration of the target analytes. In our experiments, the control line was always visible except when the CGIC strip was determined to be invalid.

### 3.2. Optimization of CGIC Strips

The analytical performance of CGIC strips was mainly affected by many parameters, such as coating antigen (rhein-BSA) concentration and antibody (mAb1F8) concentration. In this study, the effect of coating antigen concentration was optimized with negative (0 ng mL^−1^) and positive samples (200 ng mL^−1^ of rhein) using different experimental conditions (Supporting Information, Figure S1). The result presented that the color intensity of test line was deepened with the increase of the concentration of rhein-BSA. And it remained almost constant when the concentration of rhein-BSA was 1 mg mL^−1^. Meanwhile, we found that the negative and positive color contrasts were more obvious when the concentration of rhein-BSA reached 1 mg mL^−1^. The effect of antibody concentration was also optimized (Supporting Information, Figure S2). The color intensity of both test line and control line gradually increased with the increase of antibody concentration. When the concentration of mAb1F8 was higher than 3 *μ*g mL^−1^, the test line was visible. However, when it was lower than 3 *μ*g mL^−1^, the color intensity of test line was too weak for observation. Moreover, in the competitive CGIC strip, the more limited antibodies usually result in better sensitivity. Therefore, the optimal concentration of rhein-BSA and mAb1F8 was 1 mg mL^−1^ and 3 *μ*g mL^−1^, respectively.

The detection time was investigated in this study. The color intensity of test line was deepened as time goes on (Supporting Information, Figure S3). When the detection time was 6 min, the test line reached a maximum color intensity, and then, it remained constant. Thereby, the detection time was set to 6 min.

### 3.3. Evaluation of CGIC Strips

**Figure 3:**
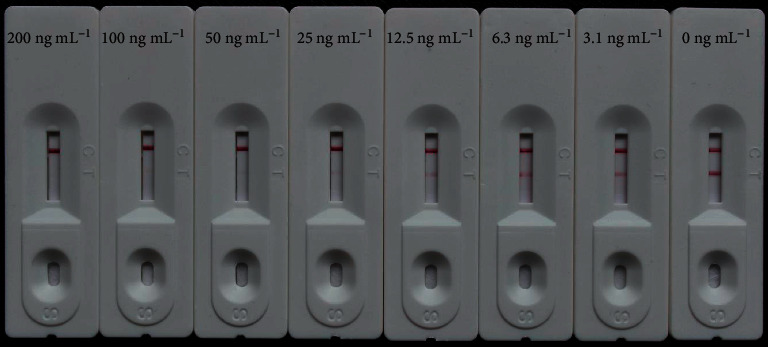
CGIC strips showing color changes corresponding to the concentrations of rhein. The cutoff value for rhein was 50 ng mL^−1^.

**Figure 4:**
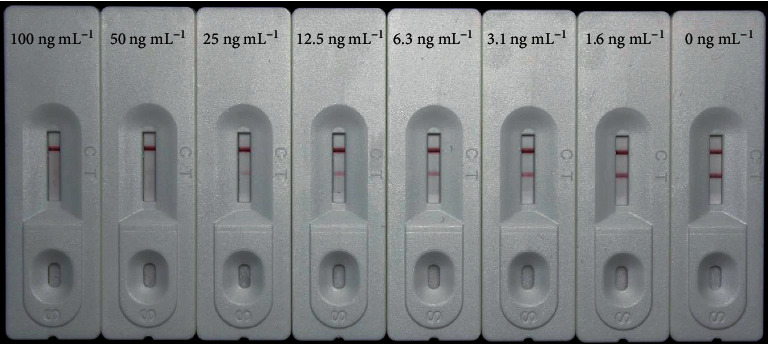
CGIC strips showing color changes corresponding to the concentrations of aloe-emodin. The cutoff value for aloe-emodin was 50 ng mL^−1^.

Stock solutions of rhein and aloe-emodin were diluted in distilled water, and gradient concentrations of rhein and aloe-emodin were tested with our CGIC strips to estimate each of their cutoff values. As shown in Figure 3, when the concentration of rhein was 50 ng mL^−1^, the color intensity of the test line was invisible. When the concentration of rhein was 100 ng mL^−1^, the color intensity of the test line was visible. Thus, we calculated that the cutoff value for rhein was 50 ng mL^−1^. Similarly, the cutoff value for aloe-emodin was also 50 ng mL^−1^ (Figure 4).

The matrix effect might cause false-positive outcomes when applying the CGIC strips to analyze rhein and aloe-emodin in real samples. In order to obtain an accurate result, dilution of extract was the most frequently used processing to decrease matrix interference and the effect of organic solvents on antibody activity [21]. The matrix effect of rhubarb extract which free of rhein and aloe-emodin was evaluated on the CGIC strips performance. The rhubarb extract was diluted with distilled water and then used to evaluate the cutoff values of rhein and aloe-emodin. The results showed that after dilution with or with more than 20-fold distilled water, the cutoff values of rhein and aloe-emodin were 50 ng mL^−1^, indicating that the matrix had no significant effect on the sensitivity of the CGIC strips.

**Figure 5:**
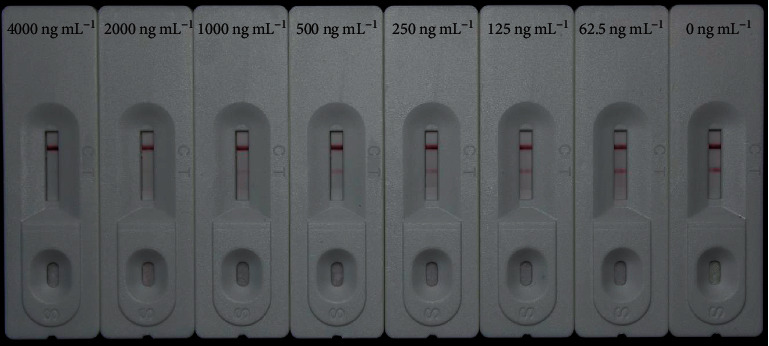
CGIC strips showing color changes corresponding to the concentrations of emodin. The cutoff value for emodin was 2000 ng mL^−1^.

**Figure 6:**
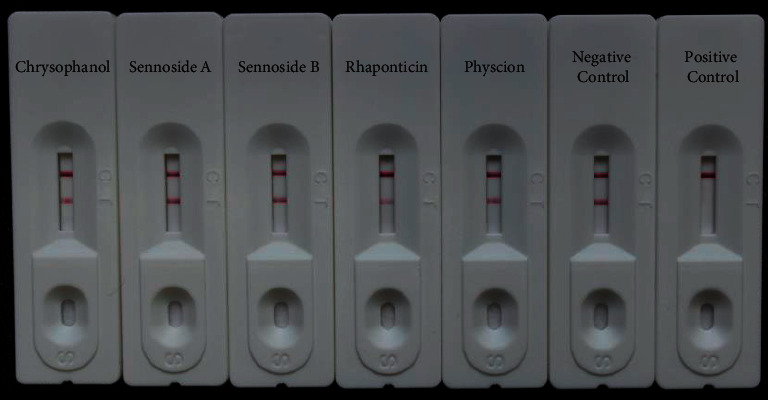
CGIC strip specificity tests of some analogues. Each standard solution was diluted in distilled water at a concentration of 5000 ng mL^−1^. The negative control was distilled water, while the positive control was rhein at a concentration of 400 ng mL^−1^. Each sample dilution was analyzed in triplicate, and the figure shows representative images.

To determine the specificity of our CGIC strips, three anthraquinones (emodin, physcion, and chrysophanol), sennoside A, sennoside B, and rhaponticin were used for evaluating of cross reactivity. The cutoff value for emodin was 2,000 ng mL^−1^ (Figure 5), which was approximately 40-fold greater than that of rhein or aloe-emodin. Moreover, no inhibition was observed for the other five compounds at a concentration of 5,000 ng mL^−1^, whereas the test line was completely inhibited by rhein or aloe-emodin (Figure 6). These results suggest that our CGIC strips are specific for rhein and aloe-emodin.

### 3.4. Analysis of Rhein and Aloe-Emodin in Crude Drug Samples

The total contents of rhein and aloe-emodin were detected by our CGIC strips after the extracted solutions of crude drug samples were appropriately diluted. The sample extract solutions of *Rheum palmatum* L. from NIFDC were diluted in distilled water by 100-, 200-, 400-, 800-, and 1600-fold and were then analyzed via our CGIC strips. No color was observed on the test line for the 100- and 200-fold dilutions, whereas the intensities of the colors of the test lines for the 400-, 800-, and 1600-fold dilutions gradually increased. These results suggest that the total concentrations of rhein and aloe-emodin in 400-fold dilutions were lower than their cutoff values. By contrast, the total concentrations of rhein and aloe-emodin in 200-fold dilutions were equal or higher than their cutoff values, which indicated that the total concentrations of rhein and aloe-emodin in the extracted solutions were between 10 and 20 *μ*g mL^−1^. Based on this estimation, the total concentrations of rhein and aloe-emodin in the other tested samples were analyzed and quantified via our CGIC strips.

In terms of HPLC analysis, the calibration curves of rhein and aloe-emodin were *Y* = 2533.4 *X* − 5.4639 (*R*^2^ = 1.0) and *Y* = 4453 *X* − 11.827 (*R*^2^ = 0.9999), respectively. In each of these equations, *Y* denotes the peak area of analyte, and *X* denotes its content (*μ*g). As shown in Table 1, the total content of rhein and aloe-emodin varied with species and companies. We can find that the total contents of rhein and aloe-emodin in official rhubarbs were ranged from 0.950 ± 0.001 mg g^−1^ to 5.717 ± 0.329 mg g^−1^, while the counterfeits were not detected. Compared the results between HPLC and CGIC strips, we can find that the results of total contents of rhein and aloe-emodin were agreed well with each other. Collectively, our findings suggested that our CGIC strips were suitable for rapid and accurate analysis of rhein and aloe-emodin contents since HPLC analysis confirmed the accuracy of our quantified results via our CGIC strips.

In addition, as shown in Table 2, the proposed CGIC strips had simpler operation, lower cost, more environmentally friendly, and better sensitivity than those in other previously reported methods.

### 3.5. Stability of CGIC Strips

The shelf life of CGIC strips was estimated under two storage conditions. As presented in Table 3, there was no change in the sensitivity of the strips within one week at 37°C and two months at 4°C. However, the cutoff values of the strips for rhein and aloe-emodin increased to 100 ng mL^−1^ after two weeks storage at 37°C and three months at 4°C.

## 4. Conclusions

In the present study, we developed a CGIC strip assay for a one-step evaluation of the total contents of two key species-specific markers, rhein and aloe-emodin, in rhubarb samples. The cutoff value of CGIC strip for rhein and aloe-emodin was 50 ng mL^−1^. For analysis of rhein and aloe-emodin in crude drug samples, the results of our CGIC strips were consistent with those of HPLC. Furthermore, the results of our CGIC strips were able to be read by the naked eye within 6 min. Storage test showed that the cutoff value for rhein and aloe-emodin remained unchanged after one week at 37°C, but increased twice after two weeks storage at 37°C and three months at 4°C. Taken together, our findings suggest that our CGIC strips may represent a novel strategy for quality control of rhubarb.

## Figures and Tables

**Table 1:** Determination of rhein and aloe-emodin contents in rhubarb samples by CGIC strip and HPLC.

Rhubarb species	Institution/company	Lot no.	Strip (mg g^−1^)	HPLC (mg g^−1^)		
				Rhein	Aloe-emodin	Total
*Rheum palmatum* L.	NIFDC	121249–201304	0.5–1.0^*a*^	0.52 ± 0.01^*b*^	0.44 ± 0.01	0.95 ± 0.01
*Rheum palmatum* L.	Suzhou Tianling CHM Co., Ltd.	20180928	4.0–8.0	3.95 ± 0.01	1.50 ± 0.01	5.45 ± 0.01
*Rheum palmatum* L.	Anguo Yuanfang CHM Co., Ltd.	20181001	4.0–8.0	4.43 ± 0.03	1.30 ± 0.01	5.72 ± 0.04
*Rheum tanguticum* Maxim. ex Balf.	NIFDC	120902–201912	1.0–2.0	0.71 ± 0.01	0.38 ± 0.01	1.09 ± 0.01
*Rheum tanguticum* Maxim. ex Balf.	Chengdu Nuanmin Co., Ltd.	20170930	4.0–8.0	3.45 ± 0.19	1.20 ± 0.03	4.65 ± 0.22
*Rheum tanguticum* Maxim. ex Balf.	Beijing Tongrentang Co., Ltd.	501003449E	4.0–8.0	4.29 ± 0.27	1.43 ± 0.06	5.72 ± 0.33
*Rheum officinale* Baill.	NIFDC	120984–201202	1.0–2.0	1.01 ± 0.01	0.61 ± 0.01	1.62 ± 0.01
*Rheum officinale* Baill.	Anguo Ruiqi CHM Co., Ltd.	20180809	4.0–8.0	3.73 ± 0.01	1.14 ± 0.01	4.86 ± 0.01
*Rumex crispus* Linn.	NIFDC	121676–201201	- - -^*c*^	—	—	—
*Rheum franzenbachii* Munt.	NIFDC	121291–201803	—	—	—	—

^*a*^Each sample was analyzed in triplicate. ^*b*^The data represented the mean ± SD. ^*c*^Not detected.

**Table 2:** Comparison of analytical performances of CGIC strips for rhein and aloe-emodin with other reported methods.

Analytes	Methods	Detection limit/cutoff value (ng mL^−1^)	Detection time (min)	Sample pretreatment	Test cost	Organic reagent	Reference
Rhein and aloe-emodin	HPLC	7.8∼17.9	20	Tedious	Expensive	Necessary	[11]
Rhein and aloe-emodin	UPLC	40∼60	3	Tedious	Expensive	Necessary	[12]
Rhein and aloe-emodin	CE	500∼1230	10	Tedious	Expensive	Necessary	[13]
Rhein and aloe-emodin	^1^H NMR	980∼2020	10	Tedious	Expensive	Necessary	[14]
Rhein and aloe-emodin	NIS	-^*a*^	64	Simple	Expensive	Unnecessary	[15]
Rhein	ELISA	20	70	Simple	Economical	Unnecessary	[17]
Rhein	QD-LCS	80	10	Simple	Economical	Unnecessary	[18]
Rhein and aloe-emodin	CGIC strips	50	6	Simple	Economical	Unnecessary	This work

^*a*^Not detected.

**Table 3:** The storage stability of CGIC strips.

Analytes	Storage conditions (°C)	Cutoff value (ng mL^−1^)					
		Storage time					
		0 day	One week	Two weeks	One month	Two months	Three months
Rhein	37	50	50	100	-^*a*^	—	—
	4	50	50	100	—	—	—

Aloe-emodin	37	50	50	50	50	50	100
	4	50	50	50	50	50	100

^*a*^Not detected.

## Data Availability

The data used to support the findings of this study are available from the corresponding author upon request.

## Supplementary Materials

Supplementary Materials Optimization of coating antigen concentration, antibody concentration, and detection time of CGIC strips. Figure S1: optimization of coating antigen concentration. Concentrations of coating antigen were 0.25, 0.5, 1.0, and 2.0 mg mL^−1^, respectively. The standard concentration: negative (0 ng mL^−1^) and positive samples (200 ng mL^−1^ of rhein). Figure S2: optimization of antibody concentration. Concentrations of antibody were 1, 2, 3, 4, and 5 *μ*g mL^−1^. Figure S3: optimization result of detection time.
