# Supplementary material for: Development of a Colloidal Gold Immunochromatographic Strip for the One-Step Evaluation of the Total Content of Rhein and Aloe-Emodin in Rhubarb
Source: Int J Anal Chem. 2022 Apr 26;2022:7067245. doi: 10.1155/2022/7067245 (PMC9064498; doi:10.1155/2022/7067245)
Supplement: Supplementary Materials — Optimization of coating antigen concentration, antibody concentration, and detection time of CGIC strips. Figure S1: optimization of coating antigen concentration. Concentrations of coating antigen were 0.25, 0.5, 1.0, and 2.0 mg mL−1, respectively. The standard concentration: negative (0 ng mL−1) and positive samples (200 ng mL−1 of rhein). Figure S2: optimization of antibody concentration. Concentrations of antibody were 1, 2, 3, 4, and 5 μg mL−1. Figure S3: optimization result of detection time. [file 7067245.f1.docx]

**Supporting Information**

**Development of a colloidal gold immunochromatographic strip for the one-step evaluating of the total content of rhein and aloe-emodin in rhubarb**

**Ping Sun, Xin-Peng Li, Jie Xin, Tao Xue, Bo Zhang*,** **Yan-Juan Liu***

School of Pharmacy, Linyi University, Linyi, 276000, P.R. China

Corresponding authors:

Bo Zhang, Phone: +86-539-7258639; Fax: +86-539-7258639; E-mail: [zhangboyxy@lyu.edu.cn](mailto:zhangboyxy@lyu.edu.cn) ;

Yan-Juan Liu, Phone: +86-539-7258639; Fax: +86-539-7258639; E-mail: liuyanjuan@lyu.edu.cn


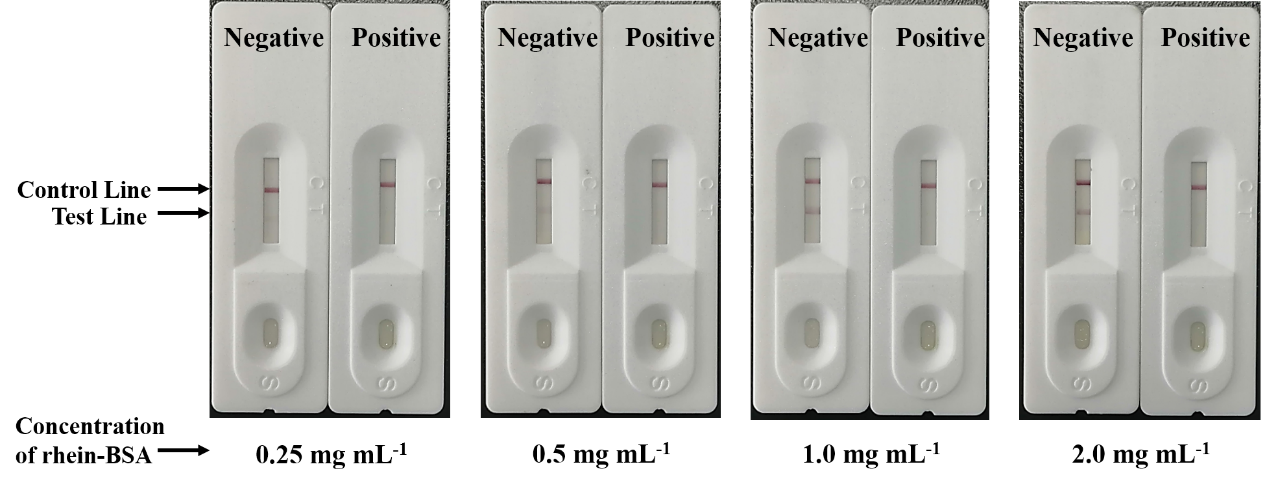


**Figure S1.** Optimization of coating antigen concentration. Concentration of coating antigen were 0.25, 0.5, 1.0, 2.0 mg mL^-1^, respectively. The standard concentration: negative (0 ng mL^-1^); positive samples (200 ng mL^-1^ of rhein).


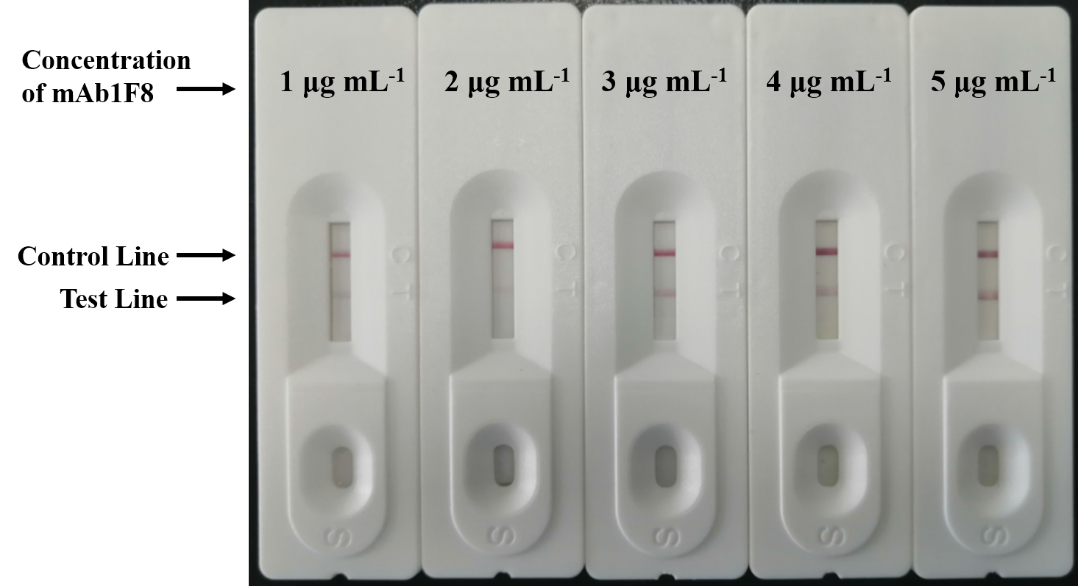


**Figure S2.** Optimization of antibody concentration. Concentration of antibody were 1, 2, 3, 4, 5 μg mL^-1^.

**
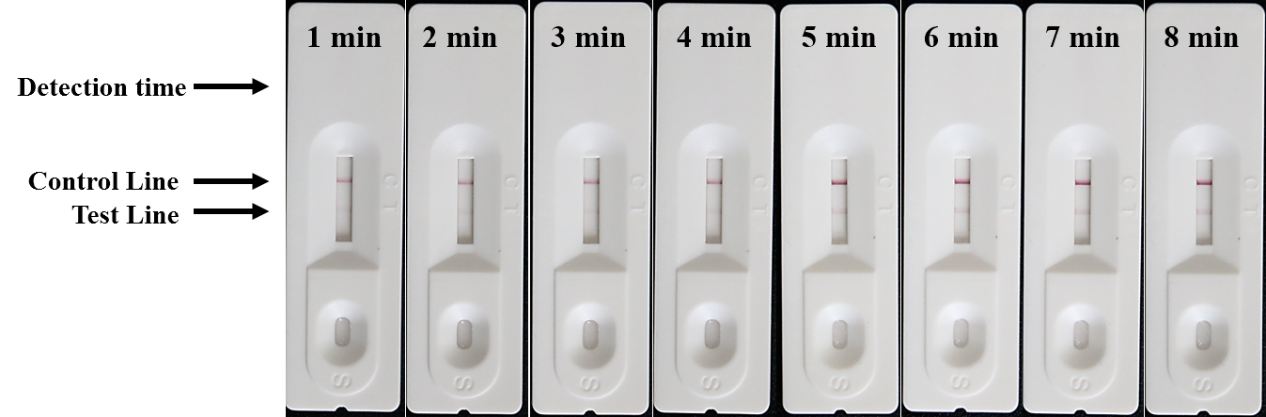
**

**Figure S3.** Optimization result of detection time.
